# Sedation and anaesthesia in pulsed-field ablation for atrial fibrillation: mechanisms, procedural requirements, and real-world practice

**DOI:** 10.1093/europace/euag127

**Published:** 2026-10-21

**Authors:** Antonio Di Monaco, Massimo Grimaldi, Stefan Hartl, Saverio Iacopino, Andreas Metzner, Usman Puar, Martin H Ruwald, Vanessa Sciacca, Philipp Sommer, Nadica Trajkovska, Maura Casciola, Maurizio Malacrida, Kars Neven

**Affiliations:** Department of Cardiology, General Regional Hospital ‘F. Miulli’, Acquaviva delle Fonti, Bari, Italy; Department of Cardiology, General Regional Hospital ‘F. Miulli’, Acquaviva delle Fonti, Bari, Italy; Department of Medicine, LUM University, Casamassima, Bari, Italy; Department of Electrophysiology, Alfried Krupp Hospital, Essen, Germany; Department of Medicine, Witten/Herdecke University, Witten, Germany; Department of Arrhythmology and Electrophysiology, Maria Cecilia Hospital, GVM Care & Research, Cotignola, Ravenna, Italy; Department of Cardiology, University Heart & Vascular Center Hamburg, Hamburg-Eppendorf, Hamburg, Germany; German Center for Cardiovascular Research (DZHK), Partner Site Hamburg/Kiel/Luebeck, Hamburg, Germany; Department of Anaesthesia and Critical Care, University Hospitals Coventry and Warwickshire, UK; Division of Electrophysiology, Department of Cardiology, Copenhagen Heart Center Rigshospitalet-Gentofte Hospital, Copenhagen, Denmark; Department of Electrophysiology, Heart and Diabetes Center NRW, Ruhr University Bochum, Bad Oeynhausen, Germany; Department of Electrophysiology, Heart and Diabetes Center NRW, Ruhr University Bochum, Bad Oeynhausen, Germany; Department of Electrophysiology, Heart and Diabetes Center NRW, Ruhr University Bochum, Bad Oeynhausen, Germany; Department of Clinical Affairs, AF Solution, Boston Scientific, Viale Forlanini 23, Milan 20134, Italy; Department of Clinical Affairs, AF Solution, Boston Scientific, Viale Forlanini 23, Milan 20134, Italy; Department of Electrophysiology, Alfried Krupp Hospital, Essen, Germany; Department of Medicine, Witten/Herdecke University, Witten, Germany

**Keywords:** Sedation, Anaesthesia, Pulsed-Field Ablation, Atrial Fibrillation, Real-World Practice

## Abstract

Pulsed-field ablation (PFA) has rapidly emerged as an important energy source for atrial fibrillation ablation, offering tissue selectivity and procedural efficiency while introducing specific anaesthetic and sedation requirements. This narrative review examines the rationale for anaesthesia and sedation during PFA, the mechanisms underlying pain, involuntary movement, and neuromuscular stimulation, and the implications of catheter design, waveform characteristics, and patient-related factors for peri-procedural management. Available evidence indicates that both deep sedation and general anaesthesia can provide safe and effective procedural conditions when supported by appropriate monitoring, airway rescue capability, and experienced multidisciplinary teams. Deep sedation may offer advantages in workflow, recovery, and patient-centred care, particularly for standard pulmonary vein isolation procedures using contemporary biphasic systems with reduced muscle activation, whereas general anaesthesia remains appropriate for complex procedures, patients with substantial comorbidity, or settings in which complete immobility and airway control are essential. Current real-world practice is highly heterogeneous across regions and institutions, reflecting differences in regulations, staffing models, procedural complexity, and local expertise rather than a universally accepted standard. Overall, anaesthetic strategy in PFA should be individualised according to patient risk profile, expected procedural demands, and device-specific stimulation burden. Further prospective comparative studies are needed to define optimal sedation depth, standardise monitoring and outcome measures, and clarify how emerging technologies may enable lighter yet still effective sedation pathways.

## Introduction

In this narrative review, we aim to provide a comprehensive analysis of the role of anaesthesia and sedation during pulsed-field ablation (PFA) for atrial fibrillation (AF), with a focus on contemporary sedation strategies, including deep sedation (DS) and general anaesthesia (GA), patient comfort, procedural safety, and optimization of clinical outcomes. We conducted this narrative review based on our clinical expertise and a targeted literature search using more than 100 keywords across major biomedical databases. Studies were selected for relevance, clinical impact, and methodological rigour, and additional references were incorporated following peer-review suggestions. Although a formal systematic protocol was not applied, our approach ensured broad coverage of key developments and evolving practices in the field.

## Why we need anaesthesia or sedation during catheter ablation in AF

Historically, anaesthesia and sedation strategies for AF catheter ablation have evolved in parallel with increasing procedural complexity, duration, and technological sophistication. In the early era of AF ablation, when focal trigger ablation and limited pulmonary vein isolation (PVI) predominated, procedures were relatively short and commonly performed under minimal or conscious sedation (CS), primarily to relieve discomfort while preserving patient responsiveness and spontaneous respiration.^1,2^ As wide antral PVI became the cornerstone of AF therapy and ablation strategies expanded to include extensive left-atrial mapping, substrate modification, and repeat procedures, workflows grew longer and technically more demanding. These developments highlighted the limitations of lighter sedation and drove a progressive shift towards deeper sedation and GA in many centres.^1,3–7^ International data from 2010 to 2019 demonstrate this global transition, with GA emerging as the most frequently used strategy by 2019 (40.5%), followed by CS (32.0%) and DS (27.5%).^1^

Currently, all these three main sedation levels—CS, DS, and GA—are used in AF ablation, with selection guided by patient characteristics, procedural complexity, legal aspects, requirements, and institutional expertise.^1^ CS is characterized by a minimally depressed level of consciousness in which patients remain responsive to verbal commands and maintain protective airway reflexes, whereas DS is associated with a more pronounced depression of consciousness, with patients responding only to repeated or painful stimulation and potentially requiring airway support.

Sedation strategy is also influenced by the ablation energy source: radiofrequency ablation (RF) has historically favoured GA, whereas cryoballoon ablation is more commonly performed under CS. In addition, marked regional variation exists, driven by legal constraints, the availability of GA services, and local practice patterns. More recently, PFA has renewed interest in DS as its fast, non-thermal energy delivery may still provoke discomfort while allowing faster recovery and avoidance of intubation, particularly in elderly patients.^7–10^ Overall, this historical progression reflects an ongoing effort to align anaesthetic strategy with evolving procedural demands rather than adherence to a single universally preferred approach. (*Table 1*).^1–15^

**Table 1:** Summary of anaesthesia trend

Era	Primary energy source	Primary sedation method	Key medication	Main goal
1998–2005	RF	Conscious sedation	Midazolam/Fentanyl	Basic comfort
2005–10	RF (increasing procedural complexity)	Deep sedation	Midazolam/Fentanyl/Propofol	Improved tolerance for longer procedures
2010–19	RF (complex left atrial ablations) Cryoballoon	General anaesthesia	Inhaled agents or Propofol/Fentanyl	Catheter stability and procedural efficiency
2020–24	High-power, short-duration RF Cryoballoon Early PFA	Deep sedation/general anaesthesia	Midazolam/Fentanyl/Propofol	Efficiency, safety, and reproducibility
≥2025	RF PFA Dual energy approaches	Deep sedation/general anaesthesia	Propofol/Fentanyl /Remimazolam	Rapid recovery, safety, workflow optimization

PFA, pulsed field ablation; RF, radiofrequency

## Patient movement, pain and discomfort, and the shared decision-making between electrophysiologist and anaesthesiologist during PFA procedures

Catheter ablation for AF is a complex procedure requiring a high degree of precision and catheter stability, even in the setting of PFA.^11,12^ Even minimal patient movement can significantly impair catheter contact, lesion quality, and procedural safety, particularly during left atrial mapping and ablation.^3,4^ Inadequate immobilization may result in prolonged procedure times, suboptimal lesion formation, increased radiation exposure, and a higher risk of an impaired safety profile.^5,6^ Pain and discomfort during AF ablation arise from multiple factors; additionally, PFA without adequate analgesia and sedation, despite its non-thermal mechanism, may induce discomfort and involuntary movements due to high-voltage electrical pulse delivery, skeletal muscle activation, and diaphragmatic or thoracic wall contraction.^9,10,13,14^ From the patient’s perspective, unmanaged pain and distress negatively affect procedural tolerance and overall experience.^7,13^ The choice between CS, DS, or GA is therefore a critical component of procedural planning and should rely on shared decision-making between electrophysiologists, anaesthesiologists, and patients.^11,12^ The decision should be based on patient-related factors (including age, comorbidities, obesity, severe pulmonary disease, sleep apnoea, and anxiety), procedural complexity, expected duration, and institutional experience.^1,6^ While electrophysiologists prioritize catheter stability, mapping accuracy, and procedural efficiency, anaesthesiologists focus on patient safety, airway management, and haemodynamic stability.^11,12^ Close interdisciplinary collaboration and standardized protocols are essential to anticipate these challenges, manage intra-procedural pain and movement effectively, and optimize procedural success and patient safety in AF ablation. (*Table 2*).^6,9,11,15^

**Table 2:** Individual priorities and primary risks in anaesthesia/sedation for AF ablation

	Main expectation/goals	Primary risks
Electrophysiologist	Catheter stability, mapping precision, procedural efficacy	Patient movement, reduced catheter contact, longer procedure times, complications
Anaesthesiologist	Airway safety, haemodynamic stability, adequate analgesia/sedation	Hypoventilation, hypotension, aspiration, over-/undersedation
Nurse/EP Lab Staff	Smooth workflows, monitoring, coordination and communication	Communication gaps, delayed response to instability or complications
Patient	Comfort, minimal pain/anxiety, rapid recovery	Pain, anxiety, poor tolerance, dissatisfaction

AF, atrial fibrillation; EP, electrophysiologist.

## Mechanisms underlying pain and movement during PFA

Pain and involuntary patient movement during PFA arise from the neuromuscular and sensory effects of high-voltage pulsed electric fields (PEFs). When properly designed, PFA delivers ultra-short, high-intensity pulses that preferentially affect myocardial tissue while sparing surrounding structures. However, skeletal muscle fibres and peripheral nerves within the electric field can also be activated, producing visible muscle contractions and nociceptive sensations. The intensity of these effects depends on many factors, including pulse amplitude, duration, number, repetition rate, catheter geometry, and proximity of tissue to the electrodes.^16,17^

At the cellular level, PEFs can elicit action potentials (APs) through multiple mechanisms. Pulses may depolarize nerve or muscle membranes without electroporation, acting directly on voltage-gated ion channels.^18^ Alternatively, reversible electroporation can create transient membrane charge imbalances that trigger APs. Understanding the mechanisms of PEF-induced nerve and muscle activation enables electrophysiology teams to anticipate patient responses and tailor sedation strategies accordingly, ensuring both safety and procedural effectiveness.

## Catheter configuration and its impact on anaesthetic Requirements

Catheter design is a key determinant of patient movement and pain during PFA, directly influencing anaesthetic requirements. Bipolar catheters confine the electric field between closely spaced electrodes, limiting unintended stimulation of adjacent nerves and skeletal muscles. This localized field reduces involuntary contractions, often allowing procedures to be performed under lighter sedation or CS protocols.^16,17^ In contrast, monopolar catheters deliver current from a single active electrode to a distant return pad, generating a broader and less predictable electric field. This increases the likelihood of activating peripheral nerves or skeletal muscles beyond the target myocardium, frequently necessitating deeper sedation or GA to maintain patient immobility and procedural safety.^18^ Several patient and procedural factors further modulate neuromuscular activation. Proximity to critical structures, including the phrenic nerve, diaphragm, or chest wall muscles, amplifies contractions and discomfort. Individual patient characteristics, such as body habitus, baseline muscle tone, and pain sensitivity, also influence responses. Procedural variables, including catheter orientation, electrode–tissue contact, and pulse train parameters, can further affect the intensity of muscle contractions.^16,17^ Clinically, these considerations may guide sedation strategies. Light sedation to CS may be sufficient when field confinement is optimized and contractions are minimal. Deeper sedation or GA may be required when higher pulse amplitudes, multiple pulse trains, or proximity to major nerves increase the likelihood of involuntary movements. However, to avoid oversimplification, it should be recognized that stimulation burden during PFA is not determined solely by whether a catheter is labelled ‘bipolar’ or ‘monopolar’. Neuromuscular activation is also influenced by electric field distribution, waveform characteristics (mono- vs. biphasic), pulse train design, electrode geometry and contact, and proximity to structures such as the phrenic nerve and diaphragm. Accordingly, sedation requirements reflect a continuum shaped by both device-specific and procedural factors rather than a deterministic bipolar–light sedation and monopolar–GA paradigm.

## Current clinical practice landscape

Currently, PFA procedures may be feasible under various sedation strategies ranging from mild CS to GA.^8,9,19–31^ However, no standardized sedation protocol has emerged, and practice patterns vary substantially across institutions and geographic regions. European, Middle Eastern, and African practices demonstrate greater variability, with electrophysiologist-driven DS protocols more commonly adopted, particularly in high-volume centres. Many European countries permit appropriately trained electrophysiologists to administer DS independently, supported by specialized nursing staff and anaesthesia consultation availability for airway emergencies.^22,23^ Importantly, the choice of sedation strategy reflected institutional practice patterns rather than patient-specific factors or standardized guidelines. Centres using DS reported shorter procedure times and fluoroscopy durations, and, more frequently, performed PVI-only procedures. Conversely, GA cases more often included electroanatomic mapping and additional lesion sets such as posterior wall isolation. European Heart Rhythm Association surveys and other multicentre assessments confirm widespread heterogeneity in sedation practices.^19–25^ Survey data indicate approximately equal adoption of GA and DS among centres offering PFA, with substantial variation between countries. This geographic variation implies that national practice patterns, regulatory environments, and healthcare system structures influence local sedation protocols. US centres have traditionally favoured GA for AF ablation procedures, while European centres have more commonly employed CS or DS strategies.^24^ The introduction of PFA has prompted some US centres to explore DS alternatives. Recent single-centre US reports demonstrate the feasibility and safety of DS for PFA when delivered with appropriate anaesthetic support.^21^ Endeavor Health (Chicago, IL) reported outcomes from 40 consecutive patients undergoing PFA under monitored anaesthesia care delivered by a certified registered nurse anaesthetist and attending anaesthesiologist. The protocol utilized propofol, dexmedetomidine, fentanyl, and midazolam (bolus and infusion at practitioner discretion) with continuous vital signs and end-tidal CO_2_ monitoring. This series achieved 100% acute PVI with no airway complications or conversions to GA. Asia-Pacific sedation practices reflect regulatory environments and healthcare system maturity levels.^29–36^ Japanese centres predominantly employ GA administered by anaesthesiologists, consistent with national medical device approval pathways that emphasize procedural standardization and comprehensive safety monitoring. In contrast, Australian practices mirror European models with greater electrophysiologist autonomy in sedation delivery. China has generated region-specific protocols that balance procedural efficiency with patient safety. Despite differences in historical practice patterns, available evidence suggests comparable clinical outcomes between GA and DS strategies when appropriate monitoring and personnel are available.^22^ Institutional protocols serve as the primary determinant of anaesthesia strategy for PFA procedures, and they reflect local resources, particularly anaesthesiology availability and staffing models. Centres with dedicated anaesthesia support may more readily adopt GA or anaesthesia-delivered DS protocols, while centres with limited anaesthesia resources may develop electrophysiologist-delivered CS pathways.^19–25^ Some choices remain constrained: individual country regulations may not allow the use of DS agents such as propofol without the presence of a full anaesthesia team. *Table 3* reports all the major studies on sedation monitoring during PFA. Despite the acknowledged role of operator preference, available studies do not provide robust multicentre data isolating operator experience as an independent predictor of anaesthesia-related complications or long-term outcomes.

**Table 3:** Sedation monitoring during pulsed field ablation

Study	Region	Sedation type	Specific agents and dosing	Sedation monitoring
EU-PORIA Registry^22^	Europe (multicentre)	Predominantly DS (*n* = 983); GA (*n* = 250)	Not reported for each centre	Institutional standard practice; staffing varied by centre
Iacopino *et al*.^8^	Europe (single centre)	DS with spontaneous respiration	Fentanyl 1.5 mcg/kg; midazolam 2 mg pre-local anaesthesia; ketamine 1 mg/kg ∼5 min before first PFA delivery	Anaesthesiologist supervision
Carboni *et al*.^9^	Europe (single centre)	Monitored anaesthesia care (MAC, with propofol) vs. GA	MAC: propofol and fentanyl GA: propofol, sevoflurane; fentanyl and remifentanil (doses per practitioner discretion)	Anaesthesia team delivered MAC or GA per operator preference
Patel *et al*.^21^	United States (Chicago, IL)	Monitored anaesthesia care/DS	Propofol, dexmedetomidine, fentanyl, midazolam (bolus and infusion at practitioner discretion)	CRNA and attending anaesthesiologist; continuous vital signs and end-tidal CO_2_ monitoring
Calvert *et al*.^26^	UK (single centre)	CS (EP-delivered)	Midazolam and fentanyl	Anaesthesiologist supervision available for conversion to GA
5S Study^27^	Europe (single centre)	DS	Propofol, midazolam, and fentanyl	Institutional standard practice for procedural sedation without GA in all patients.
Grimaldi *et al*.^28^	Europe (single centre)	DS	Midazolam, ondansetron, dexamethasone, dexmedetomidine, and remifentanil	Anaesthesiologist supervision. Standardized deep sedation with monitoring and sedation scoring
Weyand *et al*.^32^	Europe (single centre)	DS	Propofol. remifentanil, ketamine	Supervision of physician with intensive care expertise
Hiyama *et al*.^33^	Japan (single centre)	DS	Propofol, thiopental, fentanyl, and morphine	Institutional standard practice for procedural sedation without GA in all patients.
Lin *et al*.^35^	China (multicentre)	CS (89.4%)	Not reported for each centre	Anaesthesiologist supervision available for conversion to GA

AF, atrial fibrillation; DS, deep sedation; CS, conscious sedation; CRNA, certified registered nurse anaesthetist; EP, electrophysiologist; GA, general anaesthesia; MAC, monitored anaesthesia care; PFA, pulsed field ablation.

## Sedation strategies: deep sedation

DS protocols are typically based on titratable hypnotic agents, most commonly propofol (*Table 4*), with or without short-acting opioids (e.g. fentanyl; *Table 5*), and aim to achieve adequate analgesia, amnesia, patient immobility, and respiratory stability while avoiding endotracheal intubation and muscle relaxation, allowing rapid titration and recovery. In the context of PFA, DS requires specific adaptations to account for the characteristically brief but intense neuromuscular and sensory stimulation associated with pulse delivery, including anticipatory analgesia and transient deepening of sedation to ensure immobility. When implemented within standardized protocols and delivered by experienced multidisciplinary teams with appropriate monitoring and airway-rescue capabilities, DS has been shown to provide effective procedural conditions, high patient and operator satisfaction, and a favourable safety profile, supporting its role as a patient-centred alternative to GA in selected individuals. Details of the flowchart for DS and GA regimens are provided in *Figure 1*, *Figure 2* and *Figure 3*.

**Figure 1:**
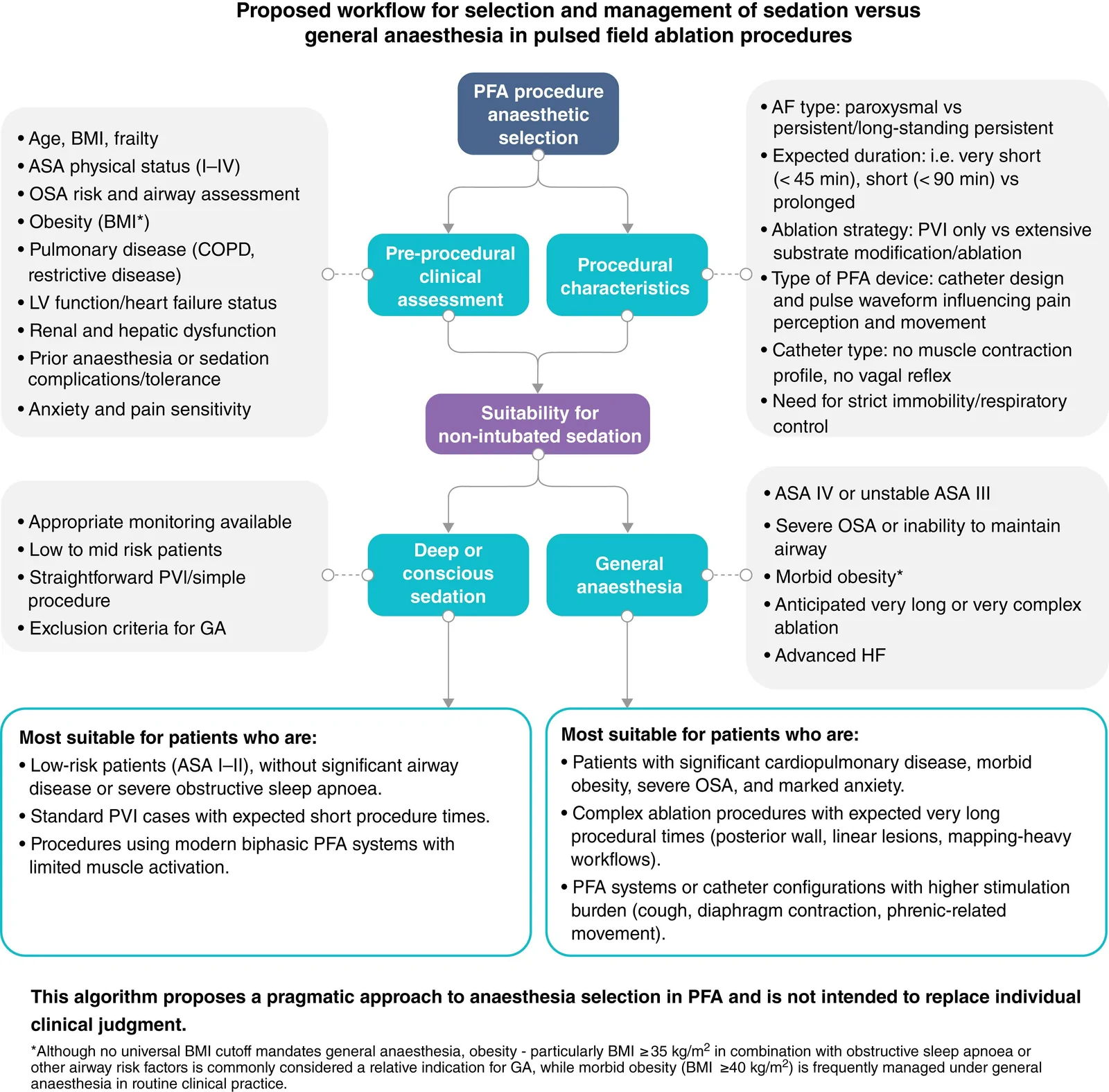
Proposed workflow for selection of anaesthesia/sedation strategy in pulsed-field ablation procedures.

**Figure 2:**
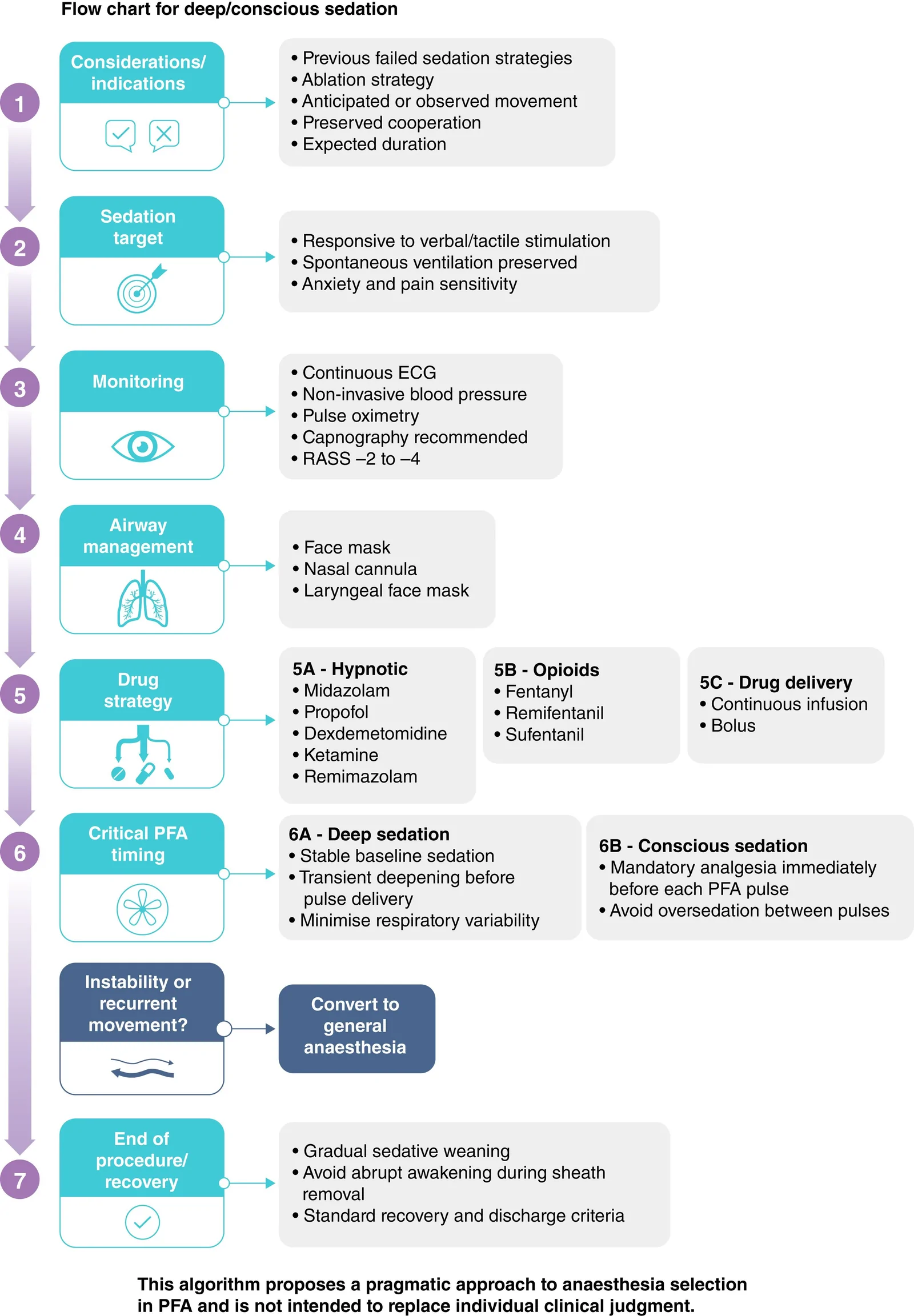
Proposed management of conscious sedation/deep sedation strategy in pulsed-field ablation procedures.

**Figure 3:**
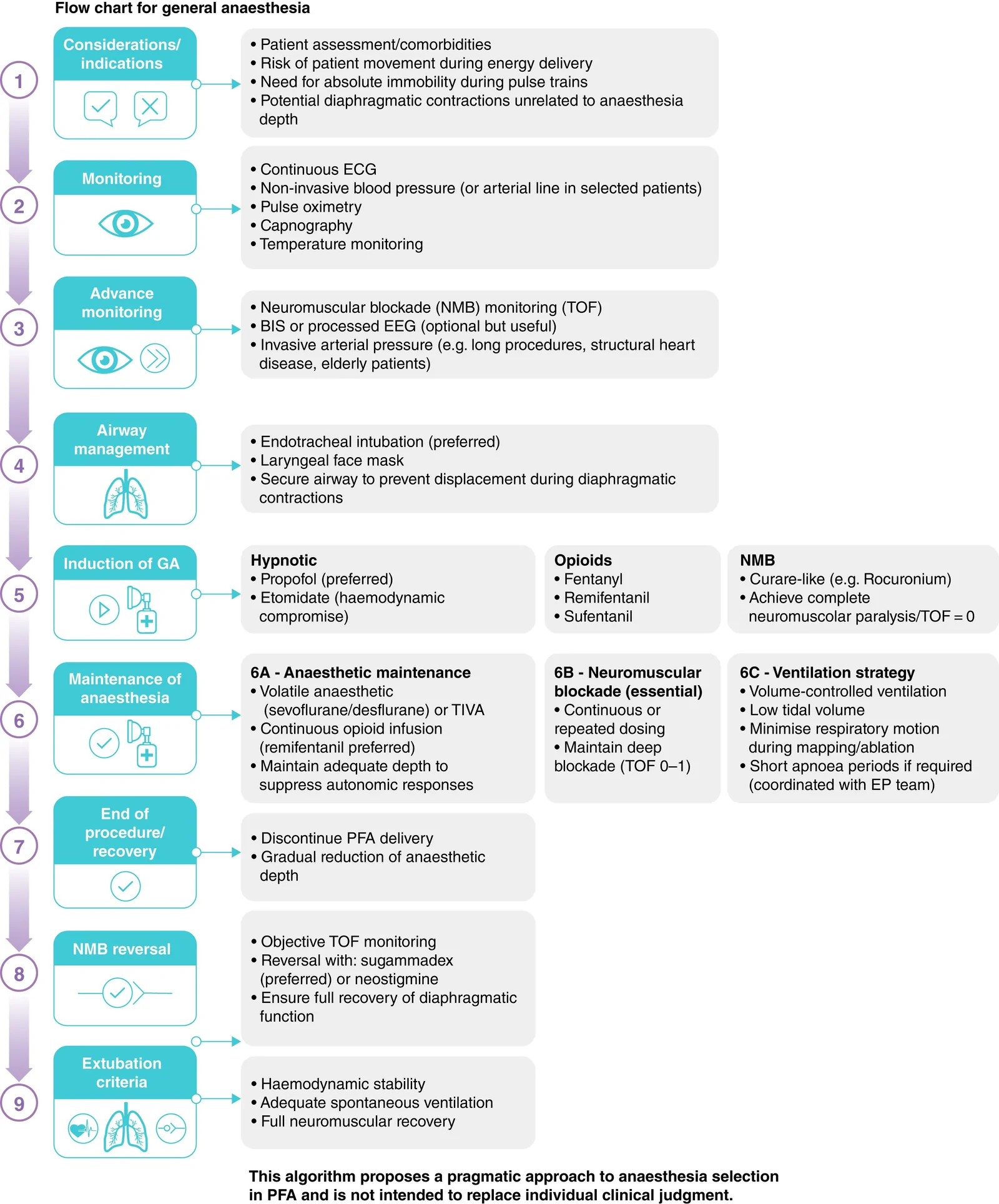
Proposed management of general anaesthesia strategy in pulsed-field ablation procedures.

**Table 4:** Hypnotic/sedative agents and key characteristics

Drug	Typical dosing range (IV)	Onset/offset	Duration	Key advantages in PFA	Key limitations/collateral effects
Midazolam	Bolus 0.5–2 mg (total 1–5 mg)	Onset 2–5 min/moderate offset	Intermediate	Anxiolysis and amnesia; simple baseline conscious sedation	Respiratory depression; prolonged effect in elderly/renal dysfunction; insufficient immobility alone
Propofol	Infusion 50–150 µg/kg/min (± bolus)	Very rapid onset (30–60 s)/rapid offset	Short	Excellent titratability; rapid deepening before PFA pulses; reliable immobility	Hypotension, apnoea, loss of airway reflexes
Dexmedetomidine	Infusion 0.2–1.0 µg/kg/h	Moderate onset/slow offset	Long (context-sensitive)	Minimal respiratory depression; opioid-sparing	Bradycardia, hypotension; slow recovery; often insufficient alone for PFA
Ketamine	Bolus 0.25–0.5 mg/kg	Rapid onset/moderate offset	Intermediate	Preserves airway reflexes; strong analgesia; sympathetic support	Emergence reactions; hypersalivation; hypertension
Remimazolam	Bolus 2.5–5 mg; infusion 1–2 mg/kg/h	Rapid onset/ultra-rapid offset	Very short	Predictable recovery; minimal accumulation; flumazenil reversible	Limited availability and experience in EP labs

EP, electrophysiologist; PFA, pulsed field ablation.

**Table 5:** Opioid analgesic and key characteristics

Drug	Typical dosing range (IV)	Onset/offset	Duration	Key advantages in PFA	Key limitations/collateral effects
Fentanyl	Bolus 25–50 µg (repeat as needed)	Rapid onset/moderate offset	Intermediate	Effective anticipatory analgesia before PFA pulses; familiar agent	Respiratory depression; accumulation with repeated boluses
Remifentanil	Infusion 0.02–0.1 µg/kg/min	Very rapid onset/ultra-rapid offset	Very short	Precise titration synchronized with PFA pulses; no accumulation	Apnoea, hypotension; requires continuous infusion and close monitoring
Sufentanil	Bolus 5–10 µg or infusion 0.1–0.3 µg/kg/h	Rapid onset/slow offset	Long	High potency; stable analgesia for repeated PFA applications	Accumulation; prolonged respiratory depression

PFA, pulsed field ablation.

### Propofol-based deep sedation

Propofol has a rapid onset, a short half-life, and requires careful cardiorespiratory monitoring due to its respiration-depressing effects and its dose-related impact on the sinus node and conduction system, potentially resulting in bradycardia and hypotension. Propofol has demonstrated its efficacy and beneficial safety profile in PFA-, cryoballoon-, and RF-based ablation in large studies, with low rates of necessary conversion to GA and a low incidence of sedation-related adverse events.^35^ Even for unipolar PFA, which causes more intense skeletal muscle contractions, propofol-based sedation was associated with a safe and effective profile.^36^ Additional bolus applications of opioids are advisable in cases of continuous pain during PFA applications. Despite its favourable characteristics, propofol should only be administered by trained and experienced personnel following structured sedation protocols.

### Non-propofol deep sedation

Although propofol is considered safe and effective when used by trained personnel, respiratory depression remains a concern. Alternatively, regimens including midazolam, ketamine, dexamethasone, remifentanil, and/or dexmedetomidine may be used in PFA procedures to achieve DS while maintaining spontaneous breathing.^8,28^ Non-propofol regimens may offer advantages in selected patients by preserving spontaneous ventilation, reducing the risk of hypotension or apnoea, and allowing for cooperative sedation; however, they may provide less predictable immobility and slower titratability, which are critical considerations during energy delivery. Consequently, non-propofol-based sedation is most often applied in carefully selected patients or as part of multimodal protocols, emphasizing the importance of individualized sedation planning and close anaesthetic monitoring. Finally, the choice of DS strategy should reflect institutional experience, institutional standards, local infrastructure, and individual patient characteristics.

## General anaesthesia approaches

GA represents the deepest level of anaesthesia, characterized by complete loss of consciousness, absence of responsiveness to painful stimuli, and the requirement for active airway management and ventilatory support.^37,38^ The full suppression of voluntary and involuntary movement, including electroporation-induced skeletal muscle contractions, historically favoured GA during early PFA procedures with monophasic waveforms.^39–41^ Meanwhile, the use of GA depends on several factors, including country-specific regulations, institutional protocols, patient characteristics, and operator preference.^22,23,25,26,42–48^  *Figure 1* and *Figure 3* illustrate the proposed workflow for GA.

### Common anaesthetic agents and monitoring requirements

GA during PFA procedures is most often induced using intravenous propofol in combination with short-acting opioids such as fentanyl or remifentanil. Depending on the PFA system and the extent of electroporation-induced muscle activity, neuromuscular blocking agents (e.g. rocuronium) have been administered to achieve complete immobilization.^35,44^ Airway management is typically achieved through endotracheal intubation or a laryngeal mask airway (LMA) with controlled mechanical ventilation to ensure stable respiratory conditions throughout the procedure. An LMA is easier to insert and less invasive compared with endotracheal intubation.^49^ However, LMA is frequently used without neuromuscular blocking agents (NMBA)s, which may increase the risk of airway reflexes. In the context of PFA, vagal nerve stimulation following pulse delivery may trigger sudden and severe laryngospasm, leading to a potentially life-threatening situation requiring urgent neuromuscular blockade and intubation.^50^ Periprocedural monitoring must be continuous throughout the administration of all anaesthetics. This includes electrocardiography, (invasive) blood pressure assessment, pulse oximetry, capnography, and temperature monitoring, in accordance with established GA safety standards.^38,45,51^ A neuromuscular-blockade monitor is required when neuromuscular blocking drugs are used, and an agent-specific anaesthetic gas monitor is required when inhalational agents are used.^46,47,51^

### Advantages versus disadvantages of general anaesthesia in PFA settings

The principal advantages of GA in the PFA setting include maximal patient comfort, complete elimination of involuntary movement, full control over respiration, and highly controlled procedural conditions for the operator,^48^ facilitating transseptal puncture and stable catheter positioning—particularly during energy application with focal PFA catheters—and enhancing 3D mapping precision.^22,24,48^ Additionally, patient management during critical intraprocedural situations may be facilitated when PFA procedures are performed under GA. These situations include pronounced vagal responses with profound bradycardia, involuntary diaphragmatic contractions, coughing, and severe hypotension, which may occur after nitroglycerine administration commonly applied prior to cavotricuspid isthmus or mitral isthmus ablation to prevent coronary artery spasm. In such scenarios, GA allows greater control of ventilation and haemodynamics, enabling rapid and effective intervention and potentially improving procedural safety. These benefits must be balanced against relevant disadvantages, including the need for secured airway management and its potential complications, dedicated anaesthesia personnel, increased resource utilization and infrastructural demands, and longer preparation and post-procedural recovery times.^22,48^ Additionally, a study comparing GA and DS using a lattice-tip PFA catheter demonstrated similar safety and efficacy, but longer laboratory occupation times with GA.^44^ Considering contemporary PFA systems that induce less pronounced muscle contractions and allow shorter procedure times, GA is increasingly applied selectively rather than as a routine standard approach.^22^ Finally, in several centres and regions, transesophageal echocardiogram-guided transseptal access when using GA represents standard practice and is considered an important safety measure. Intracardiac echocardiography may serve as an alternative imaging modality; however, its use is typically associated with a substantial increase in procedural costs.

## Role of neuromuscular blockade and muscle contraction prevention

Muscle contractions occur during PFA, arising from tissue exposure to high-voltage electric fields. Early monophasic PFA waveforms were associated with pronounced skeletal muscle activation, necessitating GA with deep neuromuscular blockade to ensure patient immobility and procedural precision.^50^ Activation of nerves innervating muscles is thought to be the most likely mechanism of muscle stimulation, rather than direct muscle fibre stimulation.^52^ Through stimulation of motor neurons innervating muscles, acetylcholine (ACh) is released at the neuromuscular junction, causing muscle fibre depolarization with a resultant increase in intracellular calcium release, leading to actin–myosin interactions and muscle contraction. The obvious consequences include diaphragmatic contraction and chest-wall movement causing coughing (also the result of a reflex arc), resulting in catheter movements, ablation-mapping shifts, and increased risk of complications such as perforation and tamponade.^53–57^ Non-depolarizing NMBAs interrupt this sequence by occupying nicotinic ACh receptors on the postsynaptic membranes of the neuromuscular junction, preventing membrane depolarization and unwanted muscle contraction during critical stages of the procedure. In contrast, contemporary biphasic waveforms have been specifically engineered to mitigate these effects.^53^ Experimental data demonstrate that short phase duration and interphase intervals, combined with longer interpulse intervals, significantly attenuate muscle contractions and pain without compromising lesion efficacy (*Figure 4*).^58^ By increasing frequency (up to 10 kHz) with short pulses (<0.5 μs), sensory, motor, and pain thresholds are increased, thus minimizing stimulation of nerves and muscles during electroporation-based treatments.^59^ These waveform refinements reduce neuromuscular excitability by limiting charge accumulation across excitable tissues, thereby improving procedural tolerability. Clinical studies and first-in-human experiences confirm that modern biphasic PFA systems exhibit substantially reduced skeletal muscle activation compared with historical monophasic platforms.^24,60^ While GA can improve procedural precision through controlled ventilation and reduced motion,^61^ there is significant evidence that DS can provide comparable outcomes.^35^ As a result, the routine use of NMBAs is no longer universally required for PFA; instead, deep-sedation protocols—often incorporating short-acting hypnotics and opioids—are increasingly favoured. It is important to note that the use of NMBAs, normally employed to facilitate optimal tracheal intubation conditions (and not for continuous endotracheal-tube tolerance during an EP procedure), risks undetected phrenic nerve injury, and protection strategies should be used.^62,63^ NMBAs may still be selectively employed as part of full anaesthesia-supported intervention in cases of excessive patient movement or discomfort, or when patient factors are not amenable to deep sedation—such as obesity, difficult airway, or significant comorbidity. GA with secured airway control remains appropriate in selected patients, such as those with severe obstructive sleep apnoea, limited cardiopulmonary reserve, or anticipated prolonged procedures, but it is no longer considered routine. It is clear that the routine use of NMB during biphasic PFA delivery is diminishing, representing a substantial departure from earlier PFA workflows that relied heavily on GA. The advent of nanosecond-waveform PFA, with its further reduction in muscle contraction and pain stimulation, will likely make CS an achievable goal.^64^

**Figure 4:**
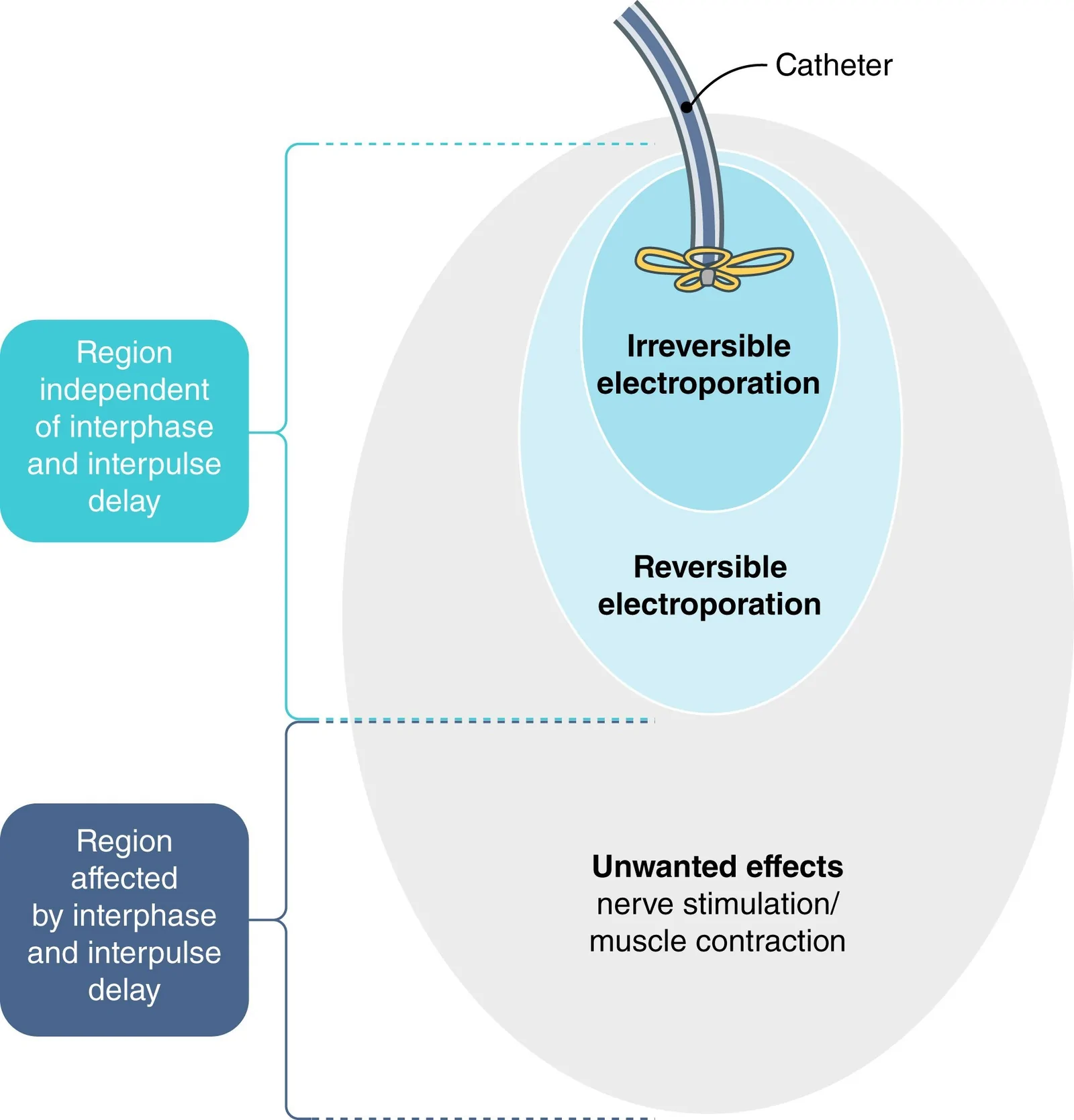
Schematic illustration of regions in and around the treatment site, showing areas dependent on and independent of the interphase (d1) and interpulse (d2) delays. The spatial extent of nerve excitation exceeds that of cell membrane electroporation, reflecting the lower activation thresholds required for neuromuscular stimulation compared with those for electroporation.^58^

## Intra-procedural management and monitoring

### Sedation strategy, airway management, and oxygenation

Airway management under DS generally relies on spontaneous ventilation with supplemental oxygen via nasal cannula or face mask, with or without an oropharyngeal airway. High-flow nasal oxygen offering CPAP may be advantageous in selected patients by maintaining oxygenation during deeper sedation required for certain phases of a PFA procedure. Continuous capnography is recommended^65^ to detect early respiratory compromise and to assist in titration of sedatives and analgesics, ensuring that the patient remains spontaneously ventilating—an essential aim during sedation. Processed EEG (e.g. BIS, PSI, entropy)^66,67^ can further assist in titration of sedatives^68,69^ and clinical recognition of sedation depth using validated scales such as the Richmond Agitation–Sedation Scale.^70^ Where deeper sedation leads to inadvertent respiratory compromise (i.e. apnoea or loss of airway), the sedationist must manage the interim period by down-titrating sedatives and maintaining oxygenation through immediate airway manoeuvres (e.g. jaw thrust, airway opening techniques, bag-valve-mask ventilation), or by using a supraglottic airway device, until spontaneous ventilation is re-established. Anaesthesia support must be readily accessible if required. The use of IV lignocaine/lidocaine 1–2 mg/kg minutes before PFA energy delivery may mitigate coughing, whether reflex-mediated or directly stimulated.^71,72^

### Pain and nociception monitoring

Despite reduced muscle contractions, electroporation remains a potent nociceptive stimulus. Pain perception during PFA has been reported even under CS, highlighting the importance of adequate analgesia in the absence of overt movement.^64^ Objective nociception monitoring may offer additional value during deeply sedated PFA procedures. Indices such as the Analgesia Nociception Index and the Nociception Level Index reliably detect nociceptive responses under anaesthesia and sedation, often preceding haemodynamic changes.^73,74^ Nociception-guided analgesia has also been associated with improved postoperative pain outcomes and reduced opioid consumption.^75^ Integrating these technologies may allow individualized analgesic titration, improving comfort while avoiding oversedation—particularly valuable for short PFA procedures.

### Haemodynamic stability

Biphasic PFA may still provoke transient autonomic responses, including brief fluctuations in blood pressure or heart rate. Continuous non-invasive or invasive haemodynamic monitoring should be selected based on patient risk profile. Balanced sedation strategies and judicious use of vasoactive agents help maintain stability, particularly in patients with structural heart disease.^24,76^

### Recovery phase considerations

One major advantage of DS for PFA is rapid recovery. Short-acting sedative and analgesic agents facilitate early awakening, neurological assessment, and mobilization. Post-procedural pain is typically mild, but systematic assessment remains important—especially when nociception-guided strategies were used intra-procedurally.

## Tailoring anaesthesia to patient and procedural needs

No universally validated algorithm exists for anaesthesia selection during PFA procedures.^22,24^ However, contemporary practice integrates multiple factors to guide individualized decision-making as reported in the proposed workflow (*Figures 1–3*). The main decision factors are:

- *The American Society of Anesthesiologists (ASA) Physical Status Classification:* ASA classification is widely used for pre-procedural risk stratification, grading patients from ASA I (healthy) to ASA VI based on systemic disease severity. In atrial fibrillation ablation, the ASA score assists in estimating anaesthetic risk and guiding the selection of CS, DS, or GA, as well as the required level of monitoring and anaesthesiology involvement. Higher ASA classes (≥III) are associated with increased susceptibility to sedation-related complications and may warrant more controlled anaesthetic approaches and closer peri-procedural surveillance.
- *Patient Comorbidities:* Pre-operative assessment should explicitly evaluate respiratory and cardiac comorbidities, as these increase risk during spontaneous-breathing sedation.^22,24,33^ Patients with significant airway risk, severe obstructive sleep apnoea, or advanced heart failure (HF) typically warrant GA for airway control and haemodynamic stability.^33,34^
- *Procedural Complexity:* More complex procedures may favour GA to ensure patient immobility and optimal procedural conditions.^8,19,22,24,28^ However, substantial variability exists across geographic regions. In contrast, shorter and more straightforward PVI procedures in low-risk patients may be suitable for DS.^8,28^
- *Catheter Type and Waveform Effects:* PFA systems vary in their stimulation profiles. Devices producing pronounced muscle contractions, cough reflex, or vagal responses necessitate deeper anaesthesia.^8,19,22,24,28^ Emerging nanosecond PFA systems that reduce muscle contractions may allow lighter sedation in selected patients, though clinical validation is ongoing.^36^

In practice, these factors interact: procedural stimulation and complexity determine the minimum sedation depth required for catheter stability, while patient comorbidity and local resources determine the safest way to deliver it. The following section summarizes the clinical evidence base that supports this individualized approach, with particular attention to outcomes and patient experience under GA and DS.

## Literature overview of clinical evidence

Clinical evidence informing anaesthesia selection for PFA is largely pragmatic rather than prescriptive. Across pivotal randomized trials^77,78^ and post-approval clinical programmes, anaesthesia was most often selected per local practice (GA or DS) rather than mandated by protocol (*Table 6*). Taken together, these programmes show that both GA and DS are feasible in randomized settings. Prospective registries and multicentre real-world series^22,89–92^ or post-approval surveillance datasets^93,94^ provide the clearest picture of contemporary sedation practice. Importantly, clinical effectiveness and low rates of major collateral injury were preserved across centres using different anaesthesia strategies, with patient selection (e.g. severe pulmonary disease, sleep apnoea) influencing the choice of GA. Single-centre and observational studies specifically addressing anaesthesia/sedation provide practical protocols and direct comparisons (*Table 6*). Early prospective single-centre studies described standardized DS regimens using midazolam/fentanyl with ketamine adjuncts,^8,92^ or midazolam/remifentanil with dexmedetomidine.^28^ More recent observational registries comparing CS (or DS without airway instrumentation) versus GA for certain PFA platforms (including over-the-wire circular and lattice-tip systems) report comparable acute isolation success, suggesting both approaches can be safe and effective when protocols, monitoring, and selection criteria are rigorously applied. From the patient perspective, DS is practical for PFA, improving workflow and patient experience without compromising safety. Positive patient feedback highlights that sedation quality strongly influences acceptance and willingness for repeat intervention.^10,20^ In the largest reported assessment of pentaspline PFA to date—evaluating both patient- and operator-reported outcomes across GA and DS strategies—both approaches were associated with uniformly high satisfaction (*Table 6*).^88^ Overall, the available literature supports a tailored approach: PFA can be performed under monitored CS/DS or under GA with airway control, with selection driven by patient comorbidity, anticipated procedural complexity, and local resources. We acknowledge that a substantial portion of the currently available clinical data refers to a single PFA technology. This largely reflects the present state of the evidence, as the majority of published clinical experience—including registry data—pertains to the most mature and widely adopted platform. These data also highlight the need for prospective, comparative studies focusing on patient experience, efficiency, different technologies, and rare adverse events, which are addressed further in subsequent sections.

**Table 6:** Literature overview of published experience focused on sedation protocol during PFA for AF

Study/Year	PFA technology/setting	Sedation/anaesthesia	Drug details/primary drug regimen	Sample size	Study type	Key findings
Grimaldi *et al*. (Europace 2023)^28^	Variable-loop biphasic PFA, PVI only	DS	Midazolam, ondansetron, dexamethasone, dexmedetomidine, remifentanil	29	Prospective, single-centre	Adequate sedation depth (RASS −1 to −5); high satisfaction; no procedural complications
Iacopino *et al*. (Europace 2023)^8^	Pentaspline PFA, PVI only	DS	low-dose fentanyl + midazolam + ketamine in spontaneous respiration	66	Prospective, single-centre	100% PVI; high patient and operator satisfaction; no major anaesthesia-related complications
Rillig *et al*. (Europace 2024)^35^	PFA with lattice-tip catheter, PVI/PVI+	GA vs. DS	GA: remifentanil infusion + bolus of propofol, then continuous infusion of both DS: bolus on fentanyl and propofol then continuous infusion of propofol	63 (23 vs. 40)	Retrospective, multicentre	Comparable acute PVI success; shorter lab occupancy with DS; low conversion to GA
Weyand *et al*. (Journal of Clinical Medicine 2024)^32^	Focal PFA, PVI/PVI+	DS	Continuous infusion of propofol and remifentanil	30	Retrospective, single-centre	High procedural efficacy; safe DS
Iacopino *et al*. (Journal of Cardiothoracic and Vascular Anesthesia 2024)^79^	Pentaspline PFA, PVI only	DS	Midazolam, fentanyl, ketamine	117	Prospective, single-centre	100% PVI; high satisfaction; effective pain control; no major sedation-related complications
Wahedi *et al*. (Journal of Cardiovascular Electrophysiology 2024)^80^	Pentaspline PFA vs. Cryo, PVI only	DS	Continuous propofol, midazolam, sufentanil	50 (PFA) + 50 (Cryo)	Prospective, single-centre	100% PVI without conversion to GA; dose of sufentanil required was significantly higher in PFA group; rate of complication similar between groups
Galuszka *et al.* (Journal of the American Heart Association 2024)^37^	Pentaspline PFA vs. Cryo vs. RF, PVI only	DS	Propofol, midazolam and fentanyl	429 (PFA) + 412 (Cryo) + 208 (RF)	Retrospective, single-centre	Total doses of propofol and fentanyl comparable between PFA and Cryo, higher than in RF.
Metzner *et al*. (Heart Rhythm 2024)^81^	PFA and RF with lattice tip catheter, PVI/PVI+	GA/DS	Intravenous propofol, midazolam and fentanyl	25 (18/7)	Prospective, single-centre	DS seemed feasible
Hirokami *et al*. (Heart Rhythm 2024)^82^	PFA and RF with lattice tip catheter, PVI/PVI+	GA/DS	GA: muscle relaxants + endotracheal intubation DS: propofol, midazolam, fentanyl	55 (15/40)	Retrospective, multicentre	100% PVI; 4 linear ablations in DS group required a switch of ablation energy
Sochorová *et al*. (Circulation 2025)^83^	Pentaspline PFA, PVI/PVI+	DS#1 vs. DS#2 vs. GA (TIVA)	DS#1 continuous remimazolam-ketamine DS#2 intermittent propofol-opioid boluses TIVA: continuous propofol-opioid with secured airway	127 (43 vs. 42 vs. 42)	Randomized, single-centre	Remimazolam-ketamine DS superior to propofol-opioid regimens (either boluses or continuous) with fewer adverse sedation events
Chen *et al*. (Interventional Journal of Cardiology 2025)^84^	Variable-diameter circular catheter, PVI/PVI+	GA/DS/CS	*Not specified*	159 (45/5/109)	Prospective, multicentre	Effectiveness comparable between CS and GA/DS, longer procedure times under CS.
Calvert *et al*. (Journal of Interventional Cardiac Electrophysiology 2025)^26^	Pentaspline PFA, PVI/PVI+	GA vs. DS	GA: Propofol CS: Midazolam + Fentanyl	23 (15 vs. 8)	Prospective, single-centre	2 patients switched from CS to GA; better post-op experience with CS; higher level of pain and anxiety with CS
Patel *et al*. (Journal of Interventional Cardiac Electrophysiology 2025)^21^	Circular loop catheter/Pentaspline PFA, PVI only	DS	Propofol, dexmedetomidine, fentanyl, midazolam	100	Retrospective, single-centre	100% PVI, no airways complications, no conversions from DS to GA
Van der Graaf *et al*. (Journal of Interventional Cardiac Electrophysiology 2025)^71^	Circular loop catheter, PVI only	GA vs. CS	GA: fentanyl, propofol, opioid continuous infusion CS: atropine, lidocaine, fentanyl, diazepam	175 (132 vs. 43)	Retrospective, single-centre	No conversion to GA; no CS patients experienced complications; 100% PVI
Nakahara *et al*. (Heart Rhythm Supplement 2025)^85^	Variable loop catheter PFA	GA vs. DS	*Not specified*	24 (12 vs. 12)	Prospective, single-centre	Minimal catheter movement with GA; 2 DS patients needed remapping due to movement
Carboni *et al.* (Europace Supplement 2025)^20^	Pentaspline PFA, PVI/PVI+	GA vs. DS	*Not specified*	478 (341 vs. 137)	Prospective, multicentre	High patient and operator satisfaction; procedural feasibility of DS across centres.
Tilz *et al*. (Europace Supplement 2025)^86^	Balloon in Basket PFA, PVI only	DS vs. DS/CS	*Not specified*	146 (91 vs. 55	Prospective, multicentre	No adverse events associated with movement in both CS/DS groups; no outcomes differences
Chun *et al*. (Europace 2025)^22^	Pentaspline PFA, PVI/PVI+	GA vs. DS	*Not specified*	1233 (250 vs. 983)	Prospective, multicentre	Fluoroscopy time shorter with DS; no differences in long term outcome
Tilz *et al*. (Heart Rhythm O2 2025)^87^	Balloon in Basket PFA, PVI/PVI+	CS	Metamizole, fentanyl, midazolam	14	Prospective, Single-centre	100% PVI; high-level of satisfaction
Sciacca *et al*. (Heart Rhythm 2025)^10^	Variable-loop catheter PFA, PVI only	DS	Propofol, Midazolam, Fentanyl	61	Prospective, Single-centre	Positive patient feedback
Compagnucci *et al.* (Heart Rhythm 2026)^88^	Pentaspline PFA, PVI/PVI+	GA vs. DS	GA: propofol with mixed strategies (desflurane, midazolam, fentanyl/remifentanil) DS: mixed approach (propofol, ketamine, midazolam, primarily fentanyl)	1188 (745 vs. 443)	Prospective, multicentre	High level of both patient- and operator-reported positive outcomes across GA and DS strategies

CS, conscious sedation; DS, deep sedation; GA, general anaesthesia; PFA, pulsed field ablation; PVI, pulmonary vein isolation; PVI+, pulmonary vein isolation with additional lesion set; RF, radiofrequency.

## Emerging trends and technological advancements

Technological innovation is rapidly reshaping anaesthetic needs in PFA. One of the most important developments is pulse modulation, which aims to reduce skeletal muscle activation by adjusting waveform shape, polarity, pulse timing, and field distribution. Particularly, the adoption of biphasic pulse configurations with optimized timing parameters has substantially reduced muscle contractions and improved procedural tolerability. These refinements are essential to incorporate into next-generation systems to meaningfully decrease involuntary contractions, thereby reducing the depth of anaesthesia required for safe and controlled ablation. Evolution of current and future PFA systems could also lead to technology-specific procedural characteristics as potential determinants in selecting the most appropriate sedation strategy. Technological integration is also improving sedation delivery. Processed EEG monitoring, advanced capnography, and nociception indices now permit finer titration of sedatives and analgesics, reducing oversedation risk.^95,96^ Alongside waveform optimization, there is increasing interest in the future role of CS-only protocols. As PFA becomes less painful and more movement-sparing, selected procedures may be performed using CS without compromising comfort, safety, or catheter stability. Early experiences in certain PFA systems, including conscious non-propofol-based sedation cohorts in recent PFA studies,^8,28,97^ suggest feasibility and good patient tolerance, although broader validation is still needed. Differences in sedation protocols may also significantly influence post-procedural recovery profiles and, consequently, the feasibility of same-day discharge following AF ablation. Optimized, fast-recovery sedation strategies could facilitate safe early mobilization and monitoring, supporting same-day discharge in carefully selected patients.^98–100^

Pharmacological innovation is another expanding frontier. Remimazolam, an ultra–short-acting benzodiazepine with predictable kinetics and rapid reversal, has emerged with the potential to offer a more haemodynamically stable and deeper sedation alternative to midazolam, and may even be comparable to propofol for EP procedures requiring titratable sedation, with the added advantage of a specific antagonist (flumazenil).^83^ Meanwhile, ketamine, although less studied in PFA, provides strong analgesia, preserves respiratory drive, and may complement low-dose opioid or benzodiazepine strategies.^79^ Together, these evolving technologies and pharmacological agents signal a future in which PFA can be performed more efficiently, more comfortably, and with reduced reliance on propofol-based deep sedation or resource-demanding GA.

## Future directions and research needs

Despite growing clinical experience, significant uncertainties remain regarding optimal sedation and anaesthesia strategies for PFA. A major gap is the lack of evidence defining the ideal anaesthesia depth, including objective thresholds for movement suppression, pain control, coughing, and haemodynamic stability. Current studies vary widely in assessment methods making comparisons across systems difficult. Standardized safety endpoints, such as hypoxia, use of necessary airway manoeuvres, hypotension, and recovery times, are also inconsistently reported. A related limitation is the absence of standardized measures for pain and movement in PFA trials. The field would benefit from consensus scoring tools that capture both procedural discomfort and electroporation-induced muscle activation. Objective tools—such as accelerometry, EMG-based movement indices, EEG-based depth monitoring and nociception, or validated pain scales—should be incorporated into future study designs to allow meaningful comparison between technologies, catheters, and sedative regimens. Further, there is a need to develop predictive models that guide anaesthesia planning based on patient characteristics (frailty, sleep apnoea, HFpEF, obesity), procedure type, and catheter configuration. Machine learning and artificial intelligence tools may help determine which patients are appropriate for CS and which require deeper support.^101,102^ Randomized trials or large-scale multicentre registries will be essential to compare sedation practices across institutions, evaluate rare adverse events, and identify best practices. Remimazolam represents a compelling candidate for future PFA workflows, but its role remains largely unexplored in electrophysiology. Finally, future PFA device innovation should integrate anaesthesia considerations directly into system design. This includes waveform refinement for minimal muscle activation, quieter or shorter pulse sequences, catheters engineered for movement-resistant stability, and real-time communication between ablation systems and anaesthetic monitors. Collaboration between engineers, electrophysiologists, and anaesthesiologists will be essential to build PFA platforms that are inherently compatible with lighter, safer, and more streamlined anaesthesia strategies.

## Key points and practical takeaways

- *Who is best suited for DS:*
  - Low-risk patients (ASA I–II), without significant airway disease or severe obstructive sleep apnoea.
  - Standard PVI cases with expected short procedure times.
  - Procedures using modern biphasic PFA systems with limited muscle activation.
- *Who is best suited for GA:*
  - Patients with significant cardiopulmonary disease, morbid obesity, severe OSA, marked anxiety.
  - Complex ablation procedures with expected very long procedural times (posterior wall, linear lesions, mapping-heavy workflows).
  - PFA systems or catheter configurations with higher stimulation burden (cough, diaphragm contraction, phrenic-related movement).
- *Sedation strategy must match expected neuromuscular stimulation:*
  - Catheters/pulse trains producing more intense contractions (diaphragmatic or chest-wall contractions) → DS with careful titration or GA.
  - Contemporary biphasic waveforms often allow DS; emerging ultra-short or nanosecond pulses may make CS viable.
- *Monitoring requirements that are non-negotiable for DS:*
  - Continuous ECG, pulse oximetry, blood-pressure monitoring.
  - Continuous capnography should be adopted to detect early hypoventilation.
  - Immediate ability to perform airway manoeuvres (jaw thrust, airway adjuncts, BVM ventilation).
  - Trained staff capable of managing inadvertent apnoea or loss of airway.
- *Monitoring requirements for GA:*
  - Full ASA-standard monitoring including capnography, neuromuscular-blockade monitoring (when NMBs are used), and temperature control.
  - Controlled ventilation via ETI or LMA, with awareness of LMA-related airway reflex risks in PFA.
- *Pain is not eliminated by reduced muscle activation:*
  - PFA remains a strong nociceptive stimulus; anticipatory analgesia is important.
  - Consider nociception-guided analgesia indices (e.g. ANI, NOL) when available.
- *Recovery considerations:*
  - DS enables rapid awakening and turnover; ideal for high-throughput PVI workflows.
  - GA may prolong recovery but ensures maximal immobility and airway control.
- *Local resources drive real-world choices and strongly influence strategy:*
  - Availability of anaesthesia support, workflow pressures, and national regulations decisively shape sedation strategy.
  - Centres with limited anaesthesia availability may rely more on electrophysiologist-directed DS workflows.
  - Centres with full anaesthesia support may use GA more liberally for efficiency and haemodynamic control.

## Conclusion

- *What is established*
  - Both DS and GA provide safe and effective procedural conditions for PFA, with comparable acute success and mid-term arrhythmia outcomes.
  - Adequate monitoring—e.g. continuous capnography and immediate airway-rescue capability—is essential for safe DS.
  - Patient-specific factors (airway, comorbidities, anxiety), procedural complexity, and institutional expertise remain central to sedation selection.
- *What is device-/centre dependent*
  - Required anaesthesia depth varies by PFA system, waveform, catheter geometry, and expected nerve/muscle stimulation.
  - Airway strategies (DS with spontaneous breathing vs. GA with LMA/ETT) differ across centres depending on staffing and regulatory frameworks.
  - Anaesthesia-delivered vs. electrophysiologist-led sedation is strongly shaped by local infrastructure, availability of anaesthesia personnel, and national norms.
- *What remains uncertain*
  - The optimal anaesthesia depth for different PFA systems is not yet defined; objective thresholds for movement suppression and analgesia are lacking.
  - No standardized metrics exist for movement burden, cough reflex, pain scores, or haemodynamic perturbations during PFA.
  - The future role of remimazolam, multimodal DS protocols, or ultra-short waveform PFA enabling true CS requires dedicated study.
  - Prospective randomized comparisons of GA vs. DS—focusing on patient experience, workflow efficiency, and rare adverse events—are still needed.

**Final approval of manuscript:** All Authors

## Data Availability

The data underlying this article will be shared on reasonable request to the corresponding author.
